# Machine Learning Identifies FLNA as a Key Molecular Target Regulating Neuronal Apoptosis after Spinal Cord Injury

**DOI:** 10.1007/s12031-025-02439-z

**Published:** 2025-11-15

**Authors:** Yingfan Pei, Yaorui Hu, Guoying Feng, Qing Xu, Shuai Zhou, Naili Zhang, Chunlei Zhang, Fei Huang, Luping Zhang

**Affiliations:** 1 Institute of Neurobiology, Binzhou Medical University, Yantai, Shandong Province China; 2 Department of Neurobiology, Cheeloo College of Medicine, Shandong University, Jinan, 250012 Shandong China; 3 Yantai Affiliated Hospital of Binzhou Medical University, Yantai, Shandong Province China

**Keywords:** Spinal cord injury (SCI), Neuronal apoptosis, Machine learning, LC‒MS/MS, PI3K/AKT signalling pathway, FLNA

## Abstract

Spinal cord injury (SCI), a traumatic type of central nervous system injury, is closely associated with neuronal apoptosis. However, the specific biomarkers and regulatory mechanisms of neuronal apoptosis in SCI patients remain unclear. In this study, we aimed to identify differentially expressed proteins (DEPs) that regulate neuronal apoptosis after SCI and reveal potential diagnostic and therapeutic targets. Spinal cord tissues were collected for LC‒MS/MS analysis at five different time points after injury. Enrichment analysis, WGCNA, random forest, support vector machine recursive feature elimination, and receiver operating characteristic (ROC) curve analysis methods were used to identify proteins and pathways associated with neuronal apoptosis. Validation was performed using a rat model and PC12 cells. A total of 351 DEPs were identified. By integrating DEPs, WGCNA, and machine learning methods, filamin A (FLNA), an apoptosis-related protein, was identified. The reliability of this finding was confirmed in the above three datasets. Spearman correlation analysis was performed to identify the top 100 proteins whose expression correlated with that of FLNA, which were then subjected to enrichment analysis. GO enrichment analysis and KEGG enrichment analysis revealed that expression of these proteins was enriched in mitochondrial oxidative phosphorylation. Western blot and qRT‒PCR analyses confirmed the upregulation of FLNA expression in a rat model of SCI. In vitro experiments revealed that silencing FLNA expression using siRNA reduced H_2_O_2_-induced apoptosis and ROS production in PC12 cells. Additionally, FLNA expression knockdown inhibited the PI3K/AKT signalling pathway. FLNA is a critical molecular target for neuronal apoptosis following SCI.

**Graphical Abstract:**

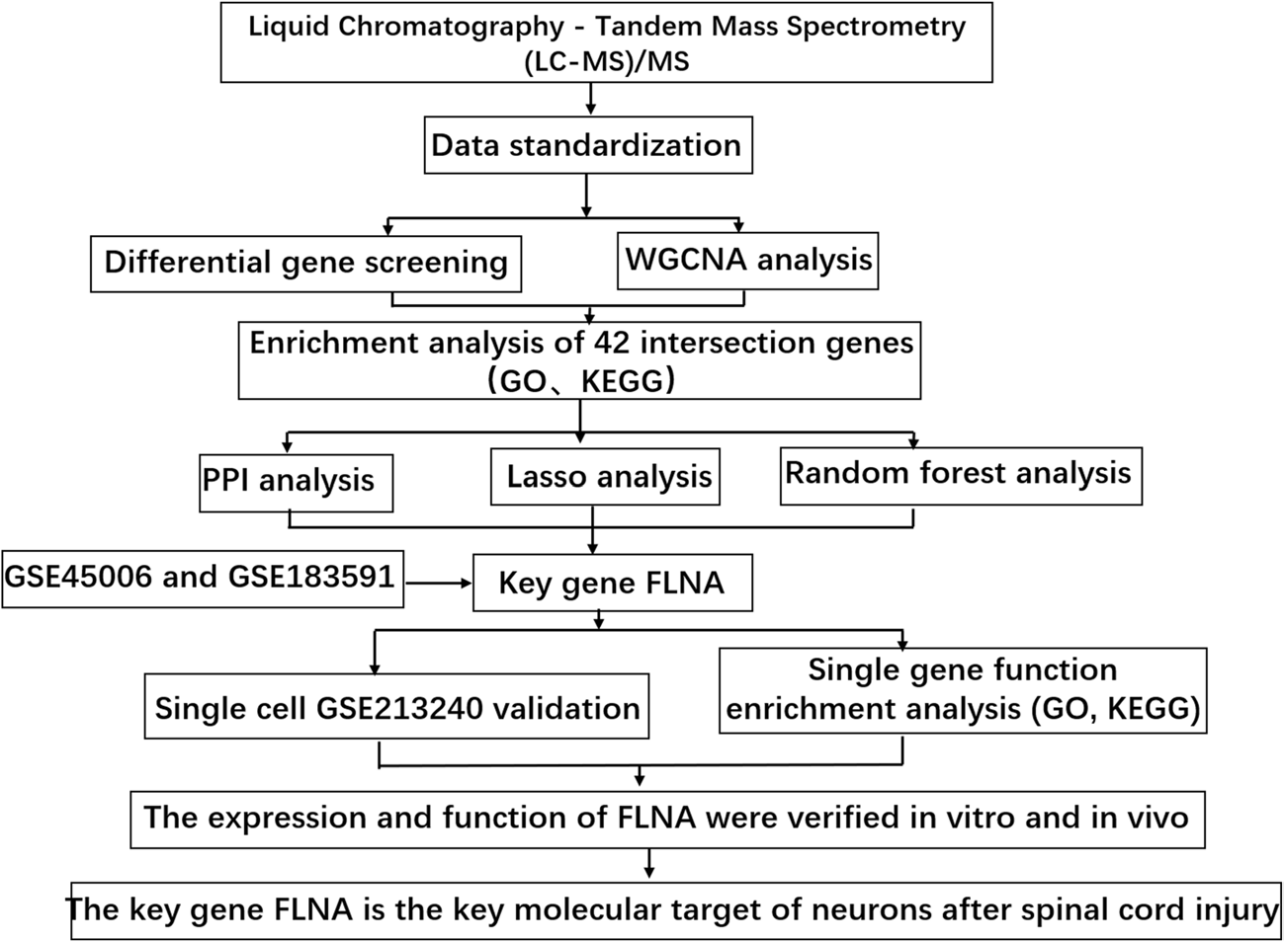

**Supplementary Information:**

The online version contains supplementary material available at 10.1007/s12031-025-02439-z.

## Introduction

Spinal cord injury (SCI) is a severe traumatic neurological disorder characterised by temporary or permanent changes in the structure and function of the spinal cord leading to neurological dysfunction below the level of injury, which may result in irreversible neurological deficits or even death (Li et al. 2025). Clinical observations have suggested that SCI patients often face long-term functional impairments, with significant motor and sensory deficits that greatly impact their quality of life and social participation (Cui et al. 2025). The pathophysiological process of SCI is highly complex, and its pathogenesis can be divided into two stages: primary injury and secondary injury (Bradbury et al. 2002; Zheng et al. 2025). Primary injury causes direct mechanical and cellular damage, triggering local ion homeostasis imbalance and the accumulation of oxidative stress products. The subsequent secondary injury cascade includes pathological processes such as damage to adjacent tissues, neuroinflammatory responses, and neuronal apoptosis mediated by ferroptosis (Zhang et al. 2025). During this process, a large amount of reactive oxygen species (ROS) are released from the injured tissue, further exacerbating the apoptotic processes. Central nervous system functional impairment caused by injury is attributed primarily to the presence of an inhibitory microenvironment and the limited regenerative capacity of the neurons themselves (Sun et al. 2024). Studies have shown that abnormal activation of mitophagy can promote excessive ROS release within cells, thereby damaging the biological functions of nerve cells (Yin et al. 2024a). Therefore, in the exploration of more effective therapeutic strategies, it is crucial to explore the molecular mechanisms and signalling pathways that regulate neuronal survival after SCI.

Advancements in proteomics have provided powerful tools for elucidating the molecular mechanisms underlying SCI. B.M. Jacobson and colleagues developed an analytical method based on liquid chromatography-tandem mass spectrometry (LC‒MS/MS) (Flardh And Jacobson 1999; Garcia-Ovejero et al. 2024). Owing to its high sensitivity and specificity, this technology has been widely applied in the field of biomedical research. LC‒MS/MS provides powerful technical support for biomarker discovery and molecular mechanism studies (Domon And Aebersold 2006). Particularly in the field of neurological disease research, LC‒MS/MS has been successfully applied to identify disease-related molecular biomarkers in various conditions, including SCI (Liu et al. 2022; Hashemi Gheinani et al. 2025; Zhou et al. 2025; Yang et al. 2022). Yao and colleagues used a label-free quantitative proteomics approach to systematically reveal differentially expressed proteins and their related signalling pathways in a rat model of spinal cord contusion (Yao et al. 2021). Pang and colleagues applied LC‒MS/MS to analyse the arachidonic acid (AA) metabolic profile in rats comprehensively during the acute phase of SCI (2, 24, and 48 h). They reported significantly elevated levels of AA metabolites such as prostaglandin E2 (PGE2) and leukotriene B4 (LTB4), along with upregulated expression of their key synthetic enzymes. These findings provide novel theoretical support for molecular targeted therapies for SCI (Pang et al. 2022).

Filamin A (FLNA) is a key cytoskeleton-related protein (Zhang 2025a). It functions mainly in mediating cross-linking actin filaments and connecting the cytoskeleton to membrane glycoproteins. This protein plays crucial regulatory roles in biological processes such as cytoskeletal remodelling, cellular shape changes, and migration via its interactions with integrins, transmembrane receptor complexes, and second messenger systems (Golushko et al. 2025). Studies have shown that FLNA regulates the proliferation dynamics of neural progenitor cells by modulating the activity of the cyclin-dependent kinase Cdk1 during the G2/M phase of the cell cycle. Loss of FLNA function not only alters cell cycle progression and proliferation rates but also disrupts cell fate determination mechanisms, leading to abnormal early differentiation (Lian et al. 2019). Moreover, FLNA phosphorylation can disrupt cell‒cell junctions and cell‒matrix adhesion. Despite these well-characterised cellular roles, the involvement of FLNA in SCI has remained largely unexplored. Most SCI-related studies have focused on inflammatory factors, apoptosis-related proteins, and neurotransmitter molecules. However, given the critical role of FLNA in cytoskeletal regulation and neural development, it has potential in SCI research as an overlooked target. The cytoskeleton is essential for maintaining the structural integrity of neurons, particularly in axonal transport, which is severely impaired after SCI. Regulation of the cytoskeleton by FLNA may influence neuronal survival and regeneration, making it a promising candidate for therapeutic intervention in SCI patients.

We completed this study in an aim to systematically screen key molecules that regulate neuronal apoptosis after SCI through the integration of multiomics data analysis and machine learning algorithms. On the basis of quantitative proteomics data at five timepoints post-SCI, bioinformatics methods were used to comprehensively analyse the differentially expressed proteins and their related signalling pathways in the acute and chronic phases of SCI. LASSO regression analysis and random forest algorithms were employed to identify SCI feature proteins, and FLNA expression reliability was further validated using the GSE183591 and GSE45006 datasets and single-cell transcriptome sequencing data. Finally, we validated the expression pattern of FLNA and the effects of FLNA expression knockdown in PC12 cells.

## Materials and Methods

### Animals

All the rat experiments were approved by the Animal Ethics Committee of Binzhou Medical University. Thirty healthy adult Sprague–Dawley rats, weighing 220–250 g, were provided by Jinan Pengyue Laboratory Animal Breeding Co., Ltd. The animals were housed under a 12-h light/dark cycle with appropriate temperature and humidity conditions. Three to five rats were housed per cage with water and food provided ad libitum.

### Rat Model of SCI

In this study, 30 eight-week-old female Sprague–Dawley rats were used. After anaesthesia, the rats were randomly divided into two groups, the sham surgery group and the SCI group, with 5 rats in each group. All SCI surgeries were performed under sterile conditions. Briefly, after the hair was shaved to fully expose the dorsal skin over the T9–T11 vertebrae, the surgical area was thoroughly disinfected with iodophor. A midline skin incision was made along the back, centred at the T10 spinous process. The T10 vertebral lamina was removed, followed by the creation of a complete transverse section at the T10 level using a sharp surgical blade (Zhao et al. 2025). Successfully induced SCI manifested as spinal cord congestion, leg swinging, tail reflex movements, and slow paralysis. The sham surgery was performed using the same procedure but without inducing spinal cord contusion. The wounds were sutured, and all the animals were housed in individual environments at 24 °C with sufficient water, food, and clean bedding. The rats underwent intermittent assisted urination twice daily until recovery of the autonomic rhythm of the bladder.

### Tissue Preparation

At 1, 3, 7, 14, and 28 days postinjury (dpi), the animals were deeply anaesthetised with sodium pentobarbital (40 mg/kg) for tissue sample collection. Spinal cord samples from the sham surgery group were collected 3 days after the sham surgery. Thirty adult Sprague–Dawley rats were randomly assigned to one of six experimental groups (sham-operated control and postoperative time points at 1, 3, 7, 14, and 28 days; *n* = 5 per group) for comprehensive molecular analyses. Each experimental group underwent three parallel detection modalities: (1) quantitative proteomic profiling, (2) Western blot validation, and (3) qRT‒PCR verification, ensuring systematic investigation at both the protein and mRNA expression levels. Animals were individually perfused with phosphate-buffered saline through the heart. Spinal cord tissues (5 mm rostral and 5 mm caudal to the injury site) was harvested and stored at − 80 °C.

### Protein Extraction

Total protein from each spinal cord tissue sample was extracted using a tissue protein extraction reagent (Thermo Fisher Scientific, Inc.). Each spinal cord sample was processed into a protein lysate and transferred to a 2.5 ml centrifuge tube. The tissue was disrupted using magnetic beads and incubated on ice for 30 min, followed by centrifugation at 12,000 × g for 10 min at 4 °C. The supernatant was collected, and the protein concentration was determined using a bicinchoninic acid (BCA) protein assay kit (Thermo Fisher Scientific, Inc.). The protein was then prepared using a 5 × laemmli protein assay kit (Thermo Fisher Scientific, Inc.) and stored at −20 °C.

### Liquid Chromatography‑Mass Spectrometry (LC‑MS)/MS and Data Processing

LC‒MS/MS experiments were performed on an EASY-nLC 1200 system. All analyses were conducted using a Q Exactive Orbitrap mass spectrometer (Thermo Fisher Scientific, Bremen, Germany) equipped with a nanoelectrospray ion source. The dried samples were dissolved in water containing 0.1% formic acid. The method was performed using two-step column separation. The analytical column used was a 10 cm EASY column (SC2003, Thermo Fisher Scientific), and the precolumn used was a 2 cm EASY chromatography column (SC001, Thermo Fisher Scientific). Peptides were eluted with a 90-min linear gradient from 4 to 100% ACN at a flow rate of 250 nL/min. The mass spectrometer was operated in positive ion mode, and spectra were acquired with a resolution of 70,000. This was followed by continuous high-collision dissociation (HCD) fragmentation of the top 10 most abundant ions. The raw proteomics data were analysed using the UniProt database. The specific database entry for *Rattus norvegicus* was identified by the accession number UP0000002494. The search parameters were as follows: the maximum tolerances for survey scans and MS/MS analysis were 10 ppm and 5 ppm, respectively. Trypsin was used as the specific enzyme, allowing a maximum of two missed cleavage sites. Carbamidomethylation of cysteine was set as a static modification, and oxidation (M) was set as a dynamic modification. The maximum false discovery rate (FDR) for peptide and protein identification was set to 1%.

### Data Processing and Differentially Expressed Protein (DEP) Identification

For the proteomics dataset, differential expression analysis was performed between the sham group and SCI group samples via t tests for rigorous statistical analysis of protein expression data. In this process, the t test function was used to precisely calculate the *P* value for each protein, which was used to assess the significance of differences in protein expression between the two groups. Moreover, on the basis of the mean changes in protein expression between the two groups, the log fold change (LogFC) for each protein was calculated. This indicator visually and quantitatively reflects the degree of changes in protein expression between the two sample groups. Proteins corresponding with DEGs were identified using the selection criteria of a *P* value < 0.05 and a |logFC|> 1. The DEGs were visualised using heatmaps and volcano plots generated with the ggplot2 and volcano packages (Martinez-Rojas et al. 2022). Additionally, to ensure the comprehensiveness and reliability of the data, batch differential protein processing was performed on the sequencing datasets GSE183591 and GSE45006 using R version 4.3.3. Under the conditions of |log2FC|> 1 and adjusted *P* < 0.05, the “limma” package was used to identify differentially expressed genes (DEGs), further validating and expanding the research findings.

### Weighted Gene Coexpression Network Analysis (WGCNA)

WGCNA was implemented. The WGCNA tool used is available on Hiplot Pro (https://hiplot.com.cn/). Hiplot Pro, a comprehensive network service platform, can be used in the analysis and visualisation of biomedical data. To explore the intrinsic correlations between module genes (module eigengenes, MEs) and SCI, Pearson correlation coefficient was chosen as the quantification metric. The Pearson correlation coefficient is a classic method used in systems biology to describe gene association patterns between different samples. This approach enables the precise identification of genes with high coexpression patterns. On the basis of the biological importance of the gene sets and the intrinsic relationship between the gene sets and phenotypes, this method aids in efficiently selecting potential candidate biomarkers or phenotypes. Additionally, it has outstanding performance in identifying candidate biomarker genes and therapeutic targets.

### Two Machine Learning Algorithms Were Used To Screen Markers

In this study, two machine learning algorithms, namely, random forest (RF) and least absolute shrinkage and selection operator (LASSO), were utilised to identify potential biomarker features. Random forest is a widely adopted machine learning approach that operates by constructing an ensemble of decision trees for feature protein selection. It is applicable to both classification and regression tasks (Tai et al. 2019; Wang et al. 2016; Ishwaran And Kogalur 2010). For the random forest analysis, the online data analysis platform Hiplot was employed. The parameters were configured as follows: the number of decision trees was set to 1000, cross-validation was performed with 10 folds, and the variable reduction rate was fixed at 1.5. These settings were determined on the basis of prior studies and preliminary experiments to ensure the reliability and stability of the analysis results. Conversely, LASSO is predominantly applied in regression analysis, variable selection, and dimensionality reduction. The primary objective of its use is to increase the predictive accuracy and interpretability of statistical models (An et al. 2023; Zanardi And Alessio 2021). In this study, the glmnet package in R was utilised to conduct cross-validation. During this process, the optimal regularisation parameter (lambda value) was carefully selected through a comprehensive evaluation of model performance across different lambda settings. This selection was crucial, as it directly influenced the effectiveness of variable selection and the overall performance of the LASSO model. Subsequently, web-based tools were leveraged to perform a cross-analysis of the outcomes from the LASSO regression and random forest analyses. By integrating the results of these two algorithms, a more comprehensive and accurate understanding of the relationships between variables was achieved. Through this cross-analysis, the key target protein FLNA was successfully identified.

### ROC Curve Analysis

To assess the performance of the key biomarkers, we used the “pROC” package in R to analyse the training set proteomics dataset as well as the GSE45006 and GSE183591 validation sets. First, for the training set, we employed relevant functions from the “pROC” package to rigorously plot the receiver operating characteristic (ROC) curve of the key biomarkers on the basis of the expression values and classification information of the biomarkers in the samples. By using precise algorithms, we calculated the sensitivity (true positive rate) and specificity (false positive rate) at different thresholds, thereby generating an accurate curve. For the GSE45006 validation set, we followed the same protocol as the training set and used the “pROC” package to plot the corresponding ROC curve on the basis of the key biomarker data and sample classifications in the validation set, ensuring that the results were comparable. Similarly, for the GSE183591 validation set, we performed the same procedure using the “pROC” package to complete the curve plotting. After the curves were plotted, we used specific functions from the “pROC” package to precisely calculate the area under the curve (AUC) for each ROC curve. The AUC is a core metric for evaluating the diagnostic performance of key biomarkers, with values ranging from 0 to 1. The closer the AUC is to 1, the better the ability to distinguish between sample categories; when the AUC is 0.5, the ability is equivalent to random guessing. By calculating the AUCs for the training set and two validation sets, we comprehensively evaluated the performance of the key biomarkers across different datasets, providing strong data support for subsequent research and application.

### Single-Cell RNA Sequencing (ScRNA-seq) Data Processing and Cell Type Identification

For single-cell genomics analysis of the GSE213240 dataset, we utilised the “Seurat” package for comprehensive exploration. For data preprocessing, strict filtering was applied. Cells with more than 6000 total expressed genes, mitochondrial gene counts above 15%, and genes expressed in fewer than 3 cells were excluded. We used log2(CPM + 1) values as the input matrix and normalised it with the “NormalizeData” function in Seurat to standardise gene expression levels across cells. To correct batch effects, the “Harmony” algorithm integrated in Seurat was employed, which effectively removed technical variation. Cell-type annotation was achieved through a multistep approach. We first compared the expression of known marker genes in our dataset with cell type-specific signatures from the Panglao DB. The “Find Clusters” function in Seurat was subsequently used with a resolution of 0.5 to identify cell clusters. Visual inspection of UMAP plots and gene expression profiles, along with references to previous studies, helped accurately label each cluster. In the analysis stage, the “Find Variable Features” function was used to identify highly variable proteins, and principal component analysis (PCA) was performed to extract key data features. For single-cell visualisation, we applied the uniform manifold approximation and projection (UMAP) method to map high-dimensional data onto a 2D plane. To identify DEGs between cell populations, we used the “Find All Markers” function with screening criteria of |log2(fold change)|> 0.25 and adjusted *p* value < 0.05. Finally, the “Dim Plot” and “Feature Plot” functions were utilised to visualise the single-cell map and gene expression patterns, respectively, facilitating the observation of the cell type distribution and specific gene expression levels (Zhang et al. 2023).

### Protein–Protein Interaction (PPI) Network Analysis

STRING (https://string-db.org/) is a database that encompasses both direct (physical) and indirect (functional) associations (Szklarczyk et al. 2021) and is used for predicting protein–protein interactions. In this study, we input a protein list into the database and selected the species "*R. norvegicus*" within the STRING database to perform the search and construct a PPI network.

### GO and KEGG Enrichment Analysis

Gene Ontology (GO) and Kyoto Encyclopedia of Genes and Genomes (KEGG) enrichment analyses are key bioinformatics approaches for analysing gene functions and biological processes. GO enrichment analysis aids in the identification of gene functions related to the dimensions of biological processes (BP), cellular components (CC), and molecular functions (MF), whereas KEGG pathway enrichment reveals the pathway associations between genes and biological processes. In this study, we carefully selected appropriate tools and databases. Specifically, the “clusterProfiler” R package was used for KEGG analysis to efficiently map the target gene set to KEGG pathways and select significantly enriched pathways. Similarly, the “Metascape” database was used for GO analysis, which integrates multiple data sources to accurately identify gene enrichment in different GO categories.

### Western Blotting

Proteins were separated via SDS‒PAGE and transferred to a PVDF membrane. After being blocked with a 10% skim milk solution for 2 h, the membrane was incubated overnight at 4 °C with specific primary antibodies. The primary antibodies used included the following: anti-GAPDH antibody (1:5000, T004, Affinity), anti-FLNA antibody (1:1000, A3738, ABclonal), anti-phosphorylated PI3K antibody (1:1000, YP0765, Immunoligy), anti-phosphorylated AKT antibody (1:1000, RMAB48852, Bioswamp), anti-PI3K antibody (1:1000, AF6241, Affinity), and anti-AKT antibody (1:500, 6,020,203–2-IG, Proteintech). The next day, the membrane was washed three times with TBST for 10 min each, and then incubated with the appropriate secondary antibody (Sino Biological Inc., Beijing, China) at room temperature for 1 h. After three washes with TBST, the protein bands were visualised via enhanced chemiluminescence (ECL) reagents (Amersham Pharmacia Biotech, Freiburg, Germany) and imaged using an enhanced chemiluminescence imaging system (Clinx, Shanghai, China). The bands were then visualised and quantified using Image Lab 3.0 software.

### Cell Culture and Transfection

The PC12 cell line is one of the most used cell lines in neuroscientific research (Wiatrak et al. 2020). In this study, PC12 cells were selected for in vitro experiments. The model of cell damage induced by treatment with H₂O₂ was used to simulate neuronal injury. PC12 cells with good growth status were seeded at a density of 1 × 10^4^ cells per well in a 96-well plate and cultured for 6 h. After the cells had adhered, they were treated with H₂O₂ for 24 h at concentrations of 10, 20, 30, 40, and 50 μmol/L. The viability of the PC12 cells was assessed using a CCK-8 assay, and a dose‒response curve was generated by plotting the viability of the cells at various concentrations. The half-maximal inhibitory concentration, corresponding to 50% cell viability, was determined and subsequently utilised to establish the H_2_O_2_-induced injury model for subsequent experiments. Our study included three groups: (1) Control group: PC12 cells cultured under standard conditions; (2) H₂O₂ group: PC12 cells treated with H₂O₂ (30 μmol/L); (3) H₂O₂ + siFLNA group: PC12 cells transfected with siFLNA and treated with H₂O₂.

PC12 cells were seeded at a density of 3 × 10^5^ cells per well in a 6-well plate. When the cell density reached 30–50%, transfection was performed using Lipofectamine 2000 with siFLNA (Obio Technology, Shanghai, China) at a concentration of 50 μM. The siRNA sequences used were as follows: forward, GAUCAAGAGUUCACAGUAATT; reverse, UUACUGUGAACUCUUGAUCTT. After 6 h incubation with the transfection reagent, the medium was replaced with normal culture medium, and after 42 h, the expression of FLNA and downstream proteins was analysed by Western blotting (WB) to verify the silencing effect and pathway expression.

### Cell Counting Kit-8 (CCK-8) Determination

The proliferation ability of PC12 cells was assessed using the CCK-8 method (Miao et al. 2023). Specifically, for the evaluation of cell viability, 1 × 10^4^ cells per well were seeded in a 96-well plate, with three wells per group. After transfection, the cells were treated with the half-maximal inhibitory concentration of H₂O₂ according to the groupings. After 24 h of cell damage, cell viability was measured using a CCK-8 assay (E—CK—A362, Elabscience, Wuhan, China).

### DHE Fluorescence Staining

Dihydroethidium (DHE) is a fluorescent probe that is commonly used to label ROS in live cells. PC12 cells in good growth conditions were subsequently seeded at 8 × 10^4^ cells per well in a 24-well plate. After the cells adhered to the culture plate, they were treated with siFLNA and H₂O₂. The medium was then replaced with medium containing 10 μM DHE working solution, and the cells were incubated at 37 °C for 30 min. Afterwards, the cells were washed with PBS, and ROS production was observed using an inverted fluorescence microscope (DMi8manual (192.0.0168), Leica, Germany).

### RNA Extraction and qRT‒PCR

Total RNA from the spinal cord tissue samples was extracted using TRIzol (Invitrogen, Carlsbad, CA, USA) according to the manufacturer’s instructions. cDNA was synthesised from 2 μg of total RNA using a high capacity cDNA reverse transcription kit (TransGen Biotech, Beijing, China). Real-time quantitative PCR was performed using SYBR Green PCR Master Mix (Vazyme Biotech Co., Ltd., Nanjing, China), with GAPDH as the internal control. Each sample was measured in triplicate. The relative expression levels were calculated using the 2^−ΔΔCt^ method and are presented as fold changes compared to the control. The sequences of primers used were as follows:

- GAPDH forward primer (5'−3')

- 5'-GGCACAGTCAAGGCTGAGAATG-3'; reverse primer (5'−3')

- 5'-ATGGTGGTGAAGACGCCAGTA-3'; FLNA forward primer (5'−3')

- 5'-CTTCCCCAGCAAGCTACAGGT-3'; reverse primer (5'−3')

- 5'-TGCCTCTCGGAAGACACCTTG-3'

### Statistical Analysis

In this study, statistical analysis was performed using GraphPad Prism 8.0.1 (GraphPad Software, La Jolla, CA, USA). Protein expression was analysed using one-way analysis of variance (ANOVA) followed by Bonferroni post hoc correction. A *p* value of less than 0.05 was considered statistically significant.

## Results

### Differentially Expressed Proteins at 5 Different Time Points after SCI

**Fig. 1:**
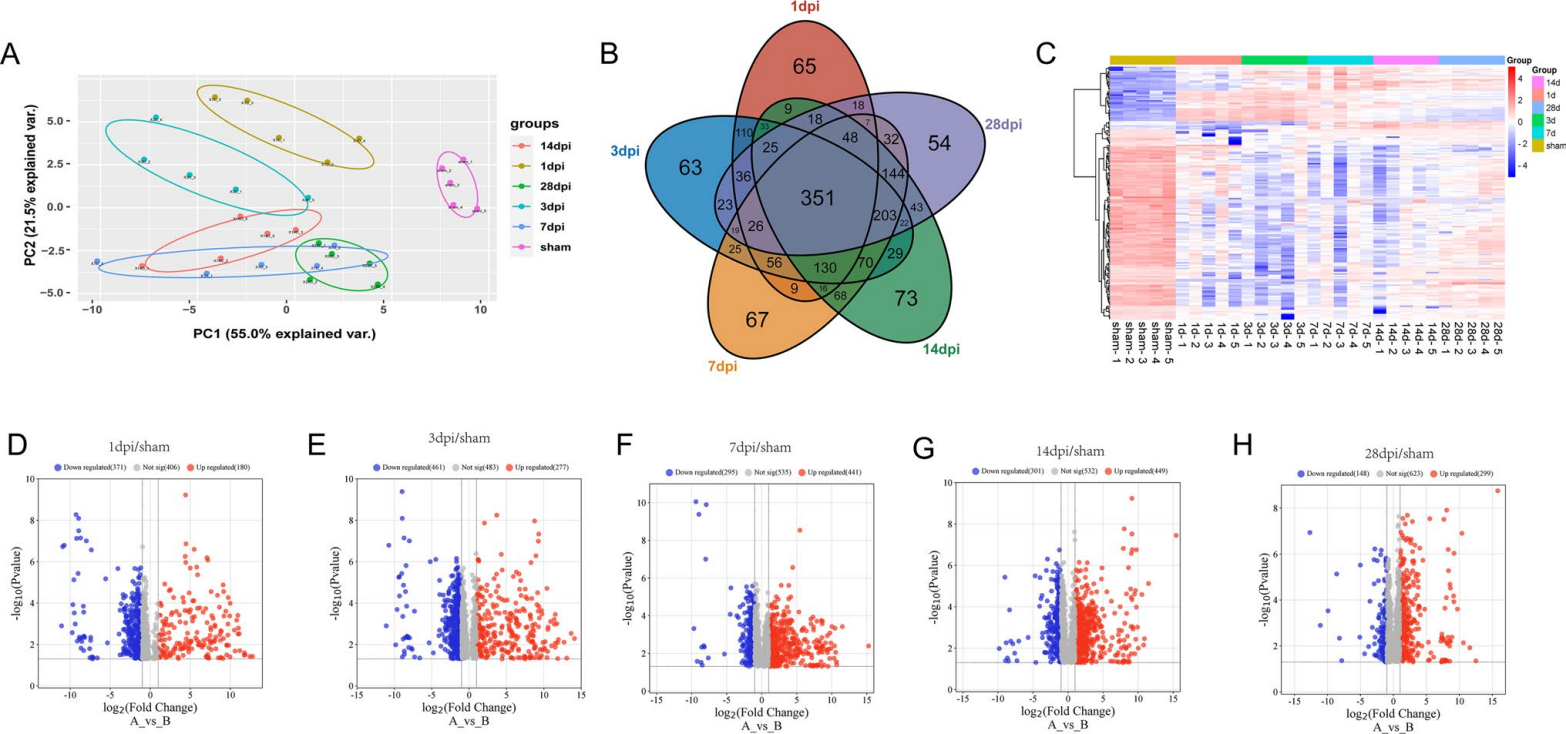
Proteomic analysis of spinal cord samples obtained at five time points after complete SCI. (**A**) Principal component analysis (PCA) revealed good intragroup consistency and intergroup differences. (**B**) Venn diagram illustrating the differences in overlapping protein expression between the 1 dpi/sham, 3 dpi/sham, 7 dpi/sham, 14 dpi/sham, and 28 dpi/sham groups. (**C**) Clustering heatmap showing the overlapping protein expression. (**D—H**) Volcano plots of the five groups

Principal component analysis (PCA) was used to verify the reasonability of the grouping of samples at different time points. The results revealed that the samples were tightly clustered within each group (e.g., high intragroup consistency at 1 dpi, 3 dpi, etc.), whereas the differences between groups were significant (e.g., separation of the SCI group from the sham-operated group), suggesting that the experimental data were reliable and could be used for subsequent differential protein analyses (Fig. 1A). Compared with those in the sham group, the number of differentially expressed proteins (DEPs) in the 1 dpi group was 957, with 1,221 in the 3 dpi group, 1,271 in the 7—dpi group, 1,282 in the 14 dpi group, and 1,069 in the 28 dpi group, with a threshold of *p* < 0.05. These DEPs are listed in Supplementary Table S1. A total of 351 differentially expressed proteins (DEPs) were identified across the five time points, with 92 proteins showing upregulated expression and 259 proteins showing downregulated expression (Fig. 1B). The heatmap (Fig. 1C) shows hierarchical clustering of the 351 overlapping DEPs across five time points to visualise the dynamics of protein expression patterns. Volcano plots (Fig. 1D-H) highlight statistically significant DEPs (|logFC|> 1, *P* < 0.05) for each time point compared with the sham group. For example, proteins involved in inflammatory responses (e.g., Ccl2 and Il6) and mitochondrial dysfunction (e.g., Ndufa4 and Cox5a) showed prominent expression upregulation at early time points (1–7 dpi), whereas proteins related to synaptic function (e.g., Syn1) showed downregulated expression in later phases (14–28 dpi). These findings align with the progression of secondary injury mechanisms, including neuroinflammation, oxidative stress, and neuronal apoptosis.

### Identification of SCI-Related Coexpressed Protein Modules

**Fig. 2:**
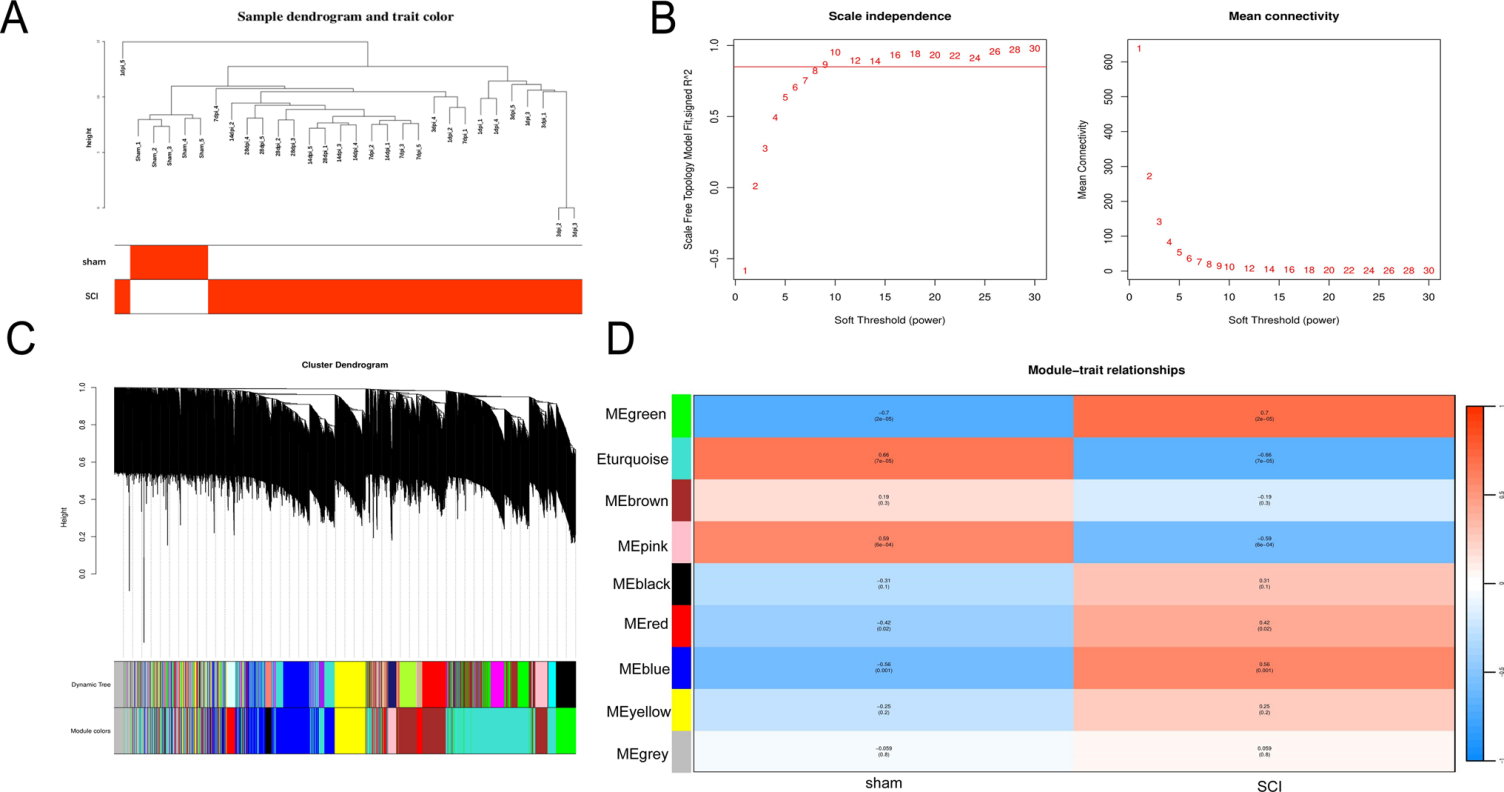
Identification of key module proteins. (**A**) The number of samples included in the analysis is presented, providing the basis for subsequent protein coexpression network construction. (**B**) Network topology analysis for various soft-threshold powers is shown, helping to determine the appropriate soft-threshold power for WGCNA. (**C**,** D**) The protein sets are divided into 9 different modules. The heatmaps in these panels illustrate the correlation between each module and the occurrence of SCI with an aim of identifying the key modules related to SCI

WGCNA facilitates the identification of disease-related modules characterised by coordinated expression patterns, thereby significantly enhancing the recognition of hub proteins (Liu et al. 2024). To construct a protein coexpression network, clustering analysis was performed on 30 samples to construct a hierarchical clustering tree. These 30 samples corresponded to the 5 time points after SCI (1, 3, 7, 14 and 28 dpi) and the sham sample group, with *n* = 5 biological replicates in each group. The results revealed that the sham surgery group was clearly separated from the SCI group (Fig. 2A), indicating that the overall effect of SCI on protein expression was significant. This tree provides a basis for grouping samples for subsequent WGCNA analyses and ensures the accuracy of the module division. A soft-threshold power of 9 was selected using the pic Soft Threshold function provided in the WGCNA package (Fig. 2B). Proteins were then classified into different modules on the basis of their expression profiles. According to average linkage clustering (Fig. 2C), the darker the colour of the module is, the stronger its correlation. After the positive correlation coefficients were analysed, the module with the strongest correlation was selected. The heatmap results indicated that the green module (r = 0.7, *P* = 0.00002) was highly significant (Fig. 2D). Therefore, the green module with the strongest significance was chosen for further analysis.

### Protein Function Enrichment Analysis

**Fig. 3:**
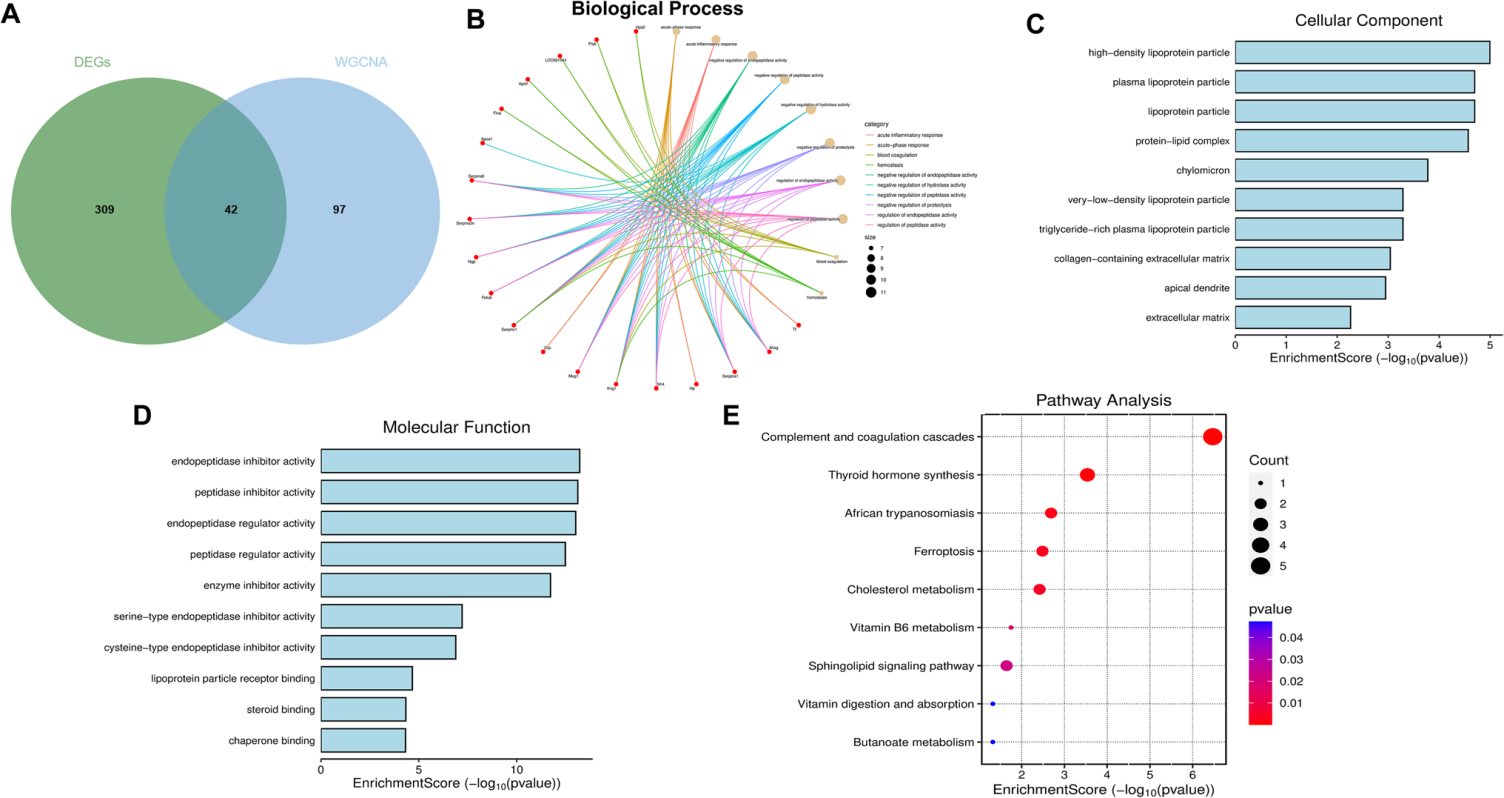
Functional enrichment analysis of the 42 intersecting proteins. (**A**) Venn diagram of the intersection between the 351 DEPs and WGCNA analysis. (**B-E**) GO and KEGG functional enrichment analysis of the 42 proteins

The 351 DEPs at the five time points intersected with the key proteins identified through WGCNA. As a result, 42 common core proteins were obtained (Fig. 3A). GO and KEGG functional enrichment analyses were subsequently performed (Fig. 3B-E). The GO enrichment analysis revealed that, in terms of biological processes, the DEPs were associated primarily with the acute inflammatory response, negative regulation of endopeptidase activity, and negative regulation of hydrolysis, among other processes. The cellular component terms were associated mainly with high-density lipoprotein particles, dendritic spines, the extracellular matrix, and lipoprotein particles. Regarding molecular functions, they were related primarily to peptidase regulator activity, steroid binding, and endopeptidase regulator activity, with the top 10 results visualised for each function. In addition, the KEGG enrichment analysis identified 9 mapped pathways, which included the complement and coagulation cascades, thyroid hormone synthesis, ferroptosis, sphingolipid signalling, cholesterol metabolism, and vitamin B6 metabolism pathways.

### Two Types of Machine Learning Screens for Key Proteins

**Fig. 4:**
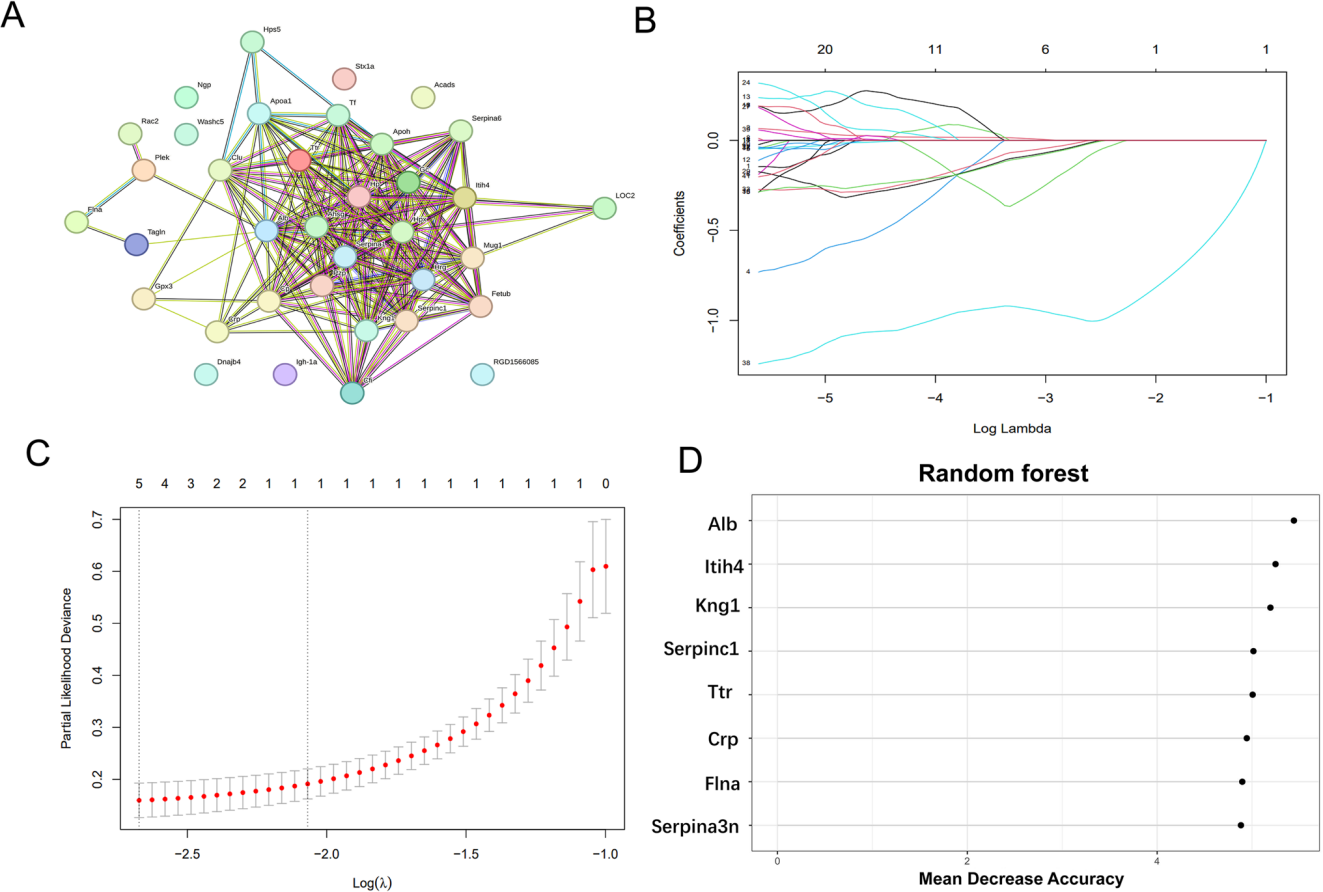
Two machine learning approaches for screening key proteins. (**A**) Venn diagram of the green module proteins and DEPs. (**B**) PPI analysis of key proteins. (**C**) LASSO regression analysis. (**D**) Identification of key proteins via the RF algorithm. DEPs: differentially expressed proteins, WGCNA: weighted gene coexpression network analysis, RF: random forest, LASSO: least absolute shrinkage and selection operator

A protein–protein interaction (PPI) network analysis was conducted using the 42 key proteins, resulting in the identification of hub proteins (Fig. 4A). Internal validation of the model was performed using the “LASSO” package, which yielded 5 feature proteins (Fig. 4B, C). The “Random Forest” package in the Hiplot online data analysis software was used to perform a random forest algorithm on the 42 differentially expressed proteins. A protein importance plot was generated with the top 8 feature proteins based on importance scores (Fig. 4D).

### FLNA Protein Expression and ROC Curve Analysis

**Fig. 5:**
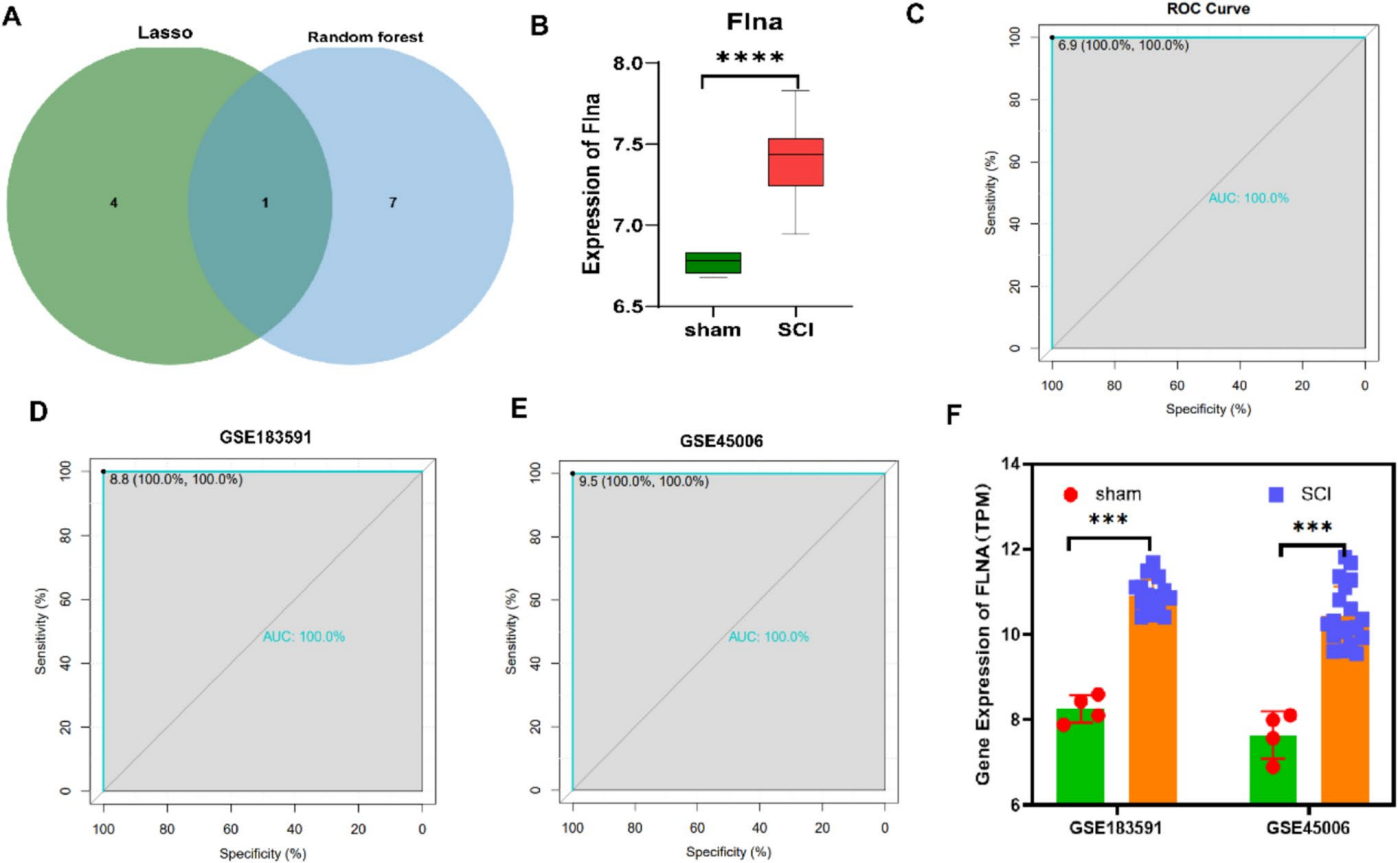
FLNA protein expression and ROC curve analysis. (**A**) Venn diagram of protein selection results generated via the LASSO and random forest algorithms. (**B**) Box plot of FLNA protein expression in the sham and SCI groups in the training set. (**C-E**) ROC curve analysis of FLNA protein expression in the training set, as well as the GSE183591 and GSE45006 datasets. (**F**) Bar chart of FLNA protein expression in the sham and SCI groups in the GSE183591 and GSE45006 datasets

The intersection of the results from the Lasso and random forest analyses is shown in Fig. 5A, revealing that FLNA is the only protein that overlaps between the two methods. The box plot (Fig. 5B) visually presents the distribution of the data, showing that FLNA expression was significantly greater in the SCI group than in the sham group (****P < 0.0001). To further assess the potential of FLNA expression as a biomarker, ROC curve analysis was performed. In the training set proteomics dataset (Fig. 5C), the AUC for FLNA expression reached 100.0%, indicating a very high diagnostic accuracy for FLNA expression in this dataset. Specifically, to validate the reliability of these results we performed further validation using different datasets, namely, GSE183591 (Fig. 5D) and GSE45006 (Fig. 5E). The results showed that in each dataset, the AUC for FLNA expression was 100.0%, further demonstrating the strong discriminatory power of the protein across different datasets. The bar chart in Fig. 5F shows FLNA protein expression in the sham and SCI groups. The chart clearly shows that the FLNA expression level in the SCI group was significantly greater than that in the sham group. Statistical analysis revealed a significant difference between the two groups (***P < 0.001), further confirming the high expression of FLNA in the SCI group. These results collectively suggest that FLNA expression may play a key role in SCI-related biological processes and has potential as a biomarker or therapeutic target.

### Single-Cell Data Analysis of FLNA Expression

**Fig. 6:**
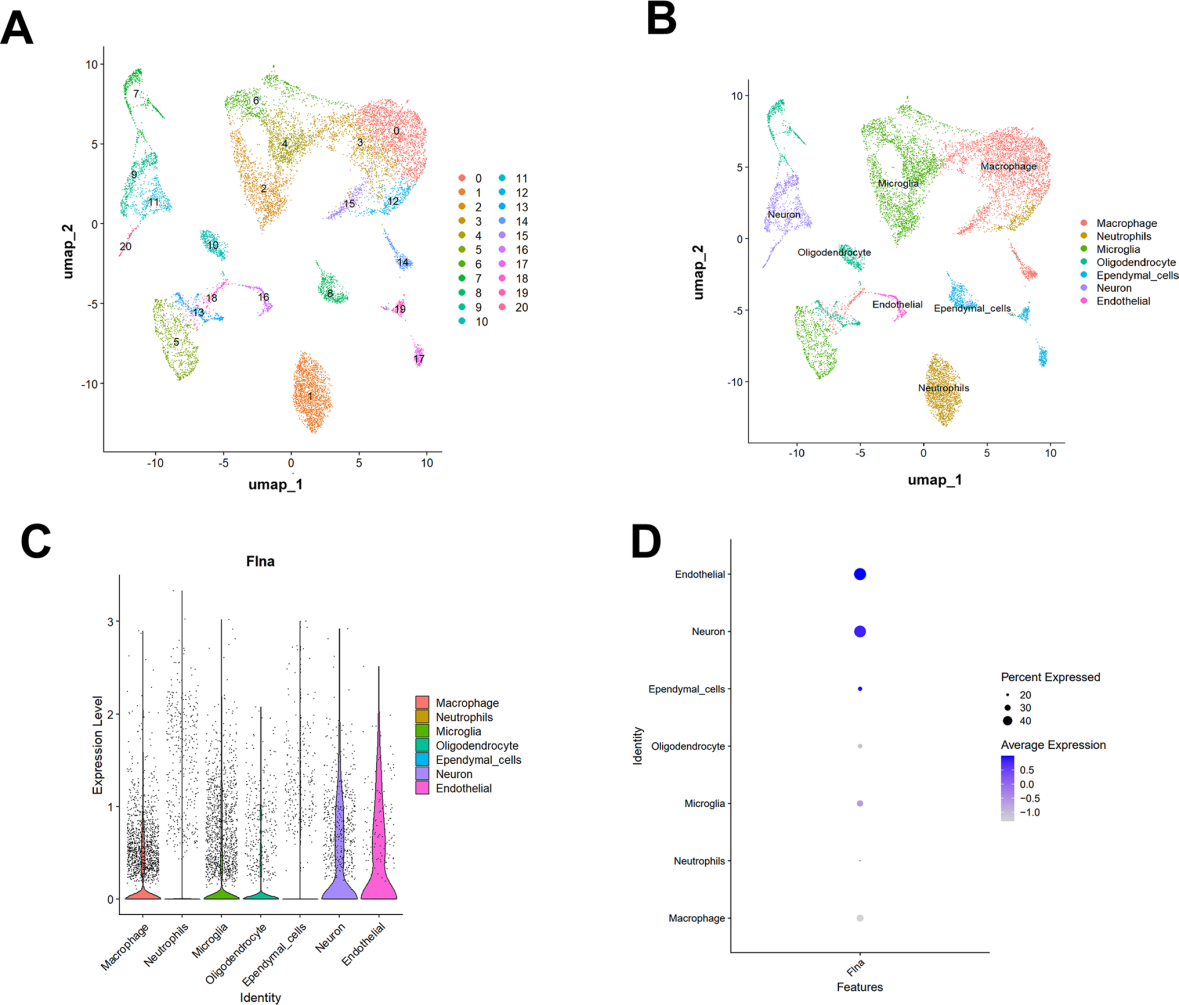
Single-cell data analysis of FLNA expression. (**A**) Single-cell clustering analysis based on the uniform manifold approximation and projection (UMAP) dimensionality reduction algorithm. (**B**) Annotation of cell clusters to identify the corresponding cell types. (**C**) Violin plot showing FLNA protein expression levels in different cell types, with the x-axis representing cell types, the y-axis representing expression levels, and different colours indicating different cell types. (**D**) Feature plot showing the percentage of cells expressing FLNA protein (percent expressed) and the average expression level (average expression) in each cell type. The size of the dots represents the percentage of expressing cells, and the colour intensity indicates the average expression level

To investigate the expression of the FLNA protein in different cell types, single-cell sequencing was used. First, single-cell clustering analysis was performed using the uniform manifold approximation and projection (UMAP) dimensionality reduction algorithm, with the results shown in Fig. 6A (single-cell clustering resulting from UMAP dimensionality reduction). The different numbers represent distinct cell clusters, and on this basis, the cell population was preliminarily divided into 20 subgroups. This clustering results laid the foundation for further analysis and served as an important framework for cell type annotation. These 20 cell clusters were subsequently annotated (Fig. 6B), and the corresponding cell types, including macrophages, neutrophils, microglia, oligodendrocytes, ependymal cells, neurons, and endothelial cells, were successfully identified. The accurate identification of these cell types provided a clear basis for the subsequent investigation of FLNA protein expression in different cellular contexts. A violin plot (Fig. 6C) was subsequently used to display expression levels of the FLNA protein across different cell types. FLNA protein expression varied significantly among cell types, with particularly high expression levels in endothelial cells and neurons and relatively low expression in some other cell types. This differential expression strongly suggests that FLNA protein expression is cell type-specific, indicating that FLNA may play different biological roles in different cell types. To more precisely reflect FLNA protein expression characteristics in various cell types, Fig. 6D presents the percentage of cells expressing FLNA and the average expression level for each cell type in the form of dots. These data provide strong support for further exploration of the function of the FLNA protein and potential mechanisms in specific cell types. In summary, these results reveal the heterogeneity of FLNA protein expression in different cell types at the single-cell level, providing important clues for further understanding the role of the FLNA protein in relevant physiological or pathological processes.

### FLNA Enrichment Analysis

**Fig. 7:**
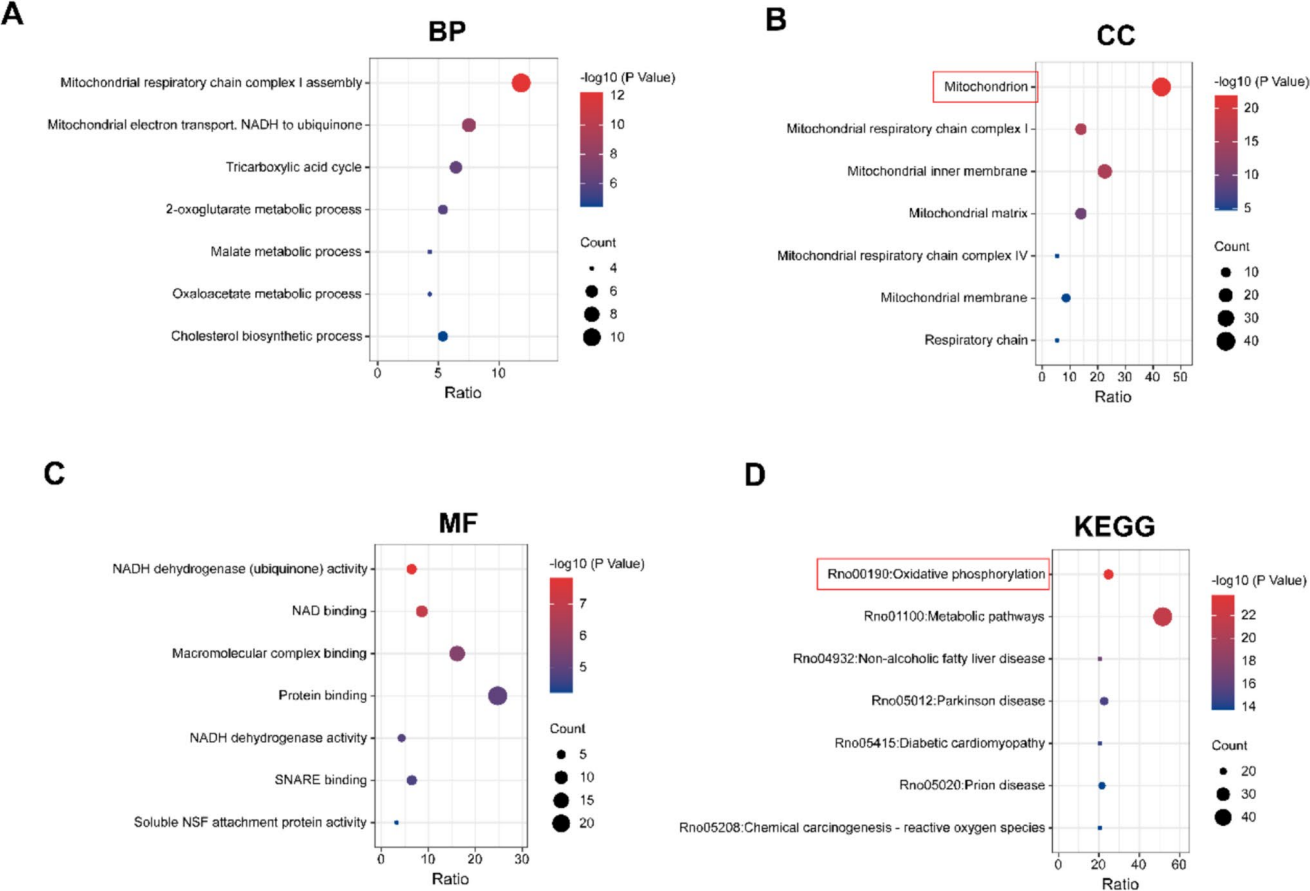
Enrichment analysis of FLNA expression. (**A-C**) Biological process (**BP**), cellular component (**CC**), and molecular function (**MF**) terms related to FLNA expression. (**D**) KEGG pathway analysis of FLNA expression

To explore the biological functions associated with FLNA, we used Pearson correlation analysis to identify the top 100 proteins whose expression was most strongly correlated with that of FLNA (*P* < 0.01). GO and KEGG analyses were subsequently performed on this gene set. The results revealed that the biological processes most closely related to FLNA expression include the assembly of mitochondrial respiratory chain complex I, mRNA processing, and protein folding (Fig. 7A). Furthermore, the cellular components most highly associated with FLNA expression are located in the cytoplasm and mitochondria (Fig. 7B). The molecular functions most highly associated with FLNA expression are protein binding and homeoprotein binding (Fig. 7C). The signalling pathways most closely related to FLNA expression include oxidative phosphorylation, neurodegeneration, and apoptosis (Fig. 7D). These findings suggest that FLNA expression plays an important regulatory role in the pathophysiological process of SCI, potentially influencing the progression of SCI by regulating mitochondrial function.

### Knocking Down FlNA Expression Can Reduce Oxidative Stress Damage in PC12 Cells

**Fig. 8:**
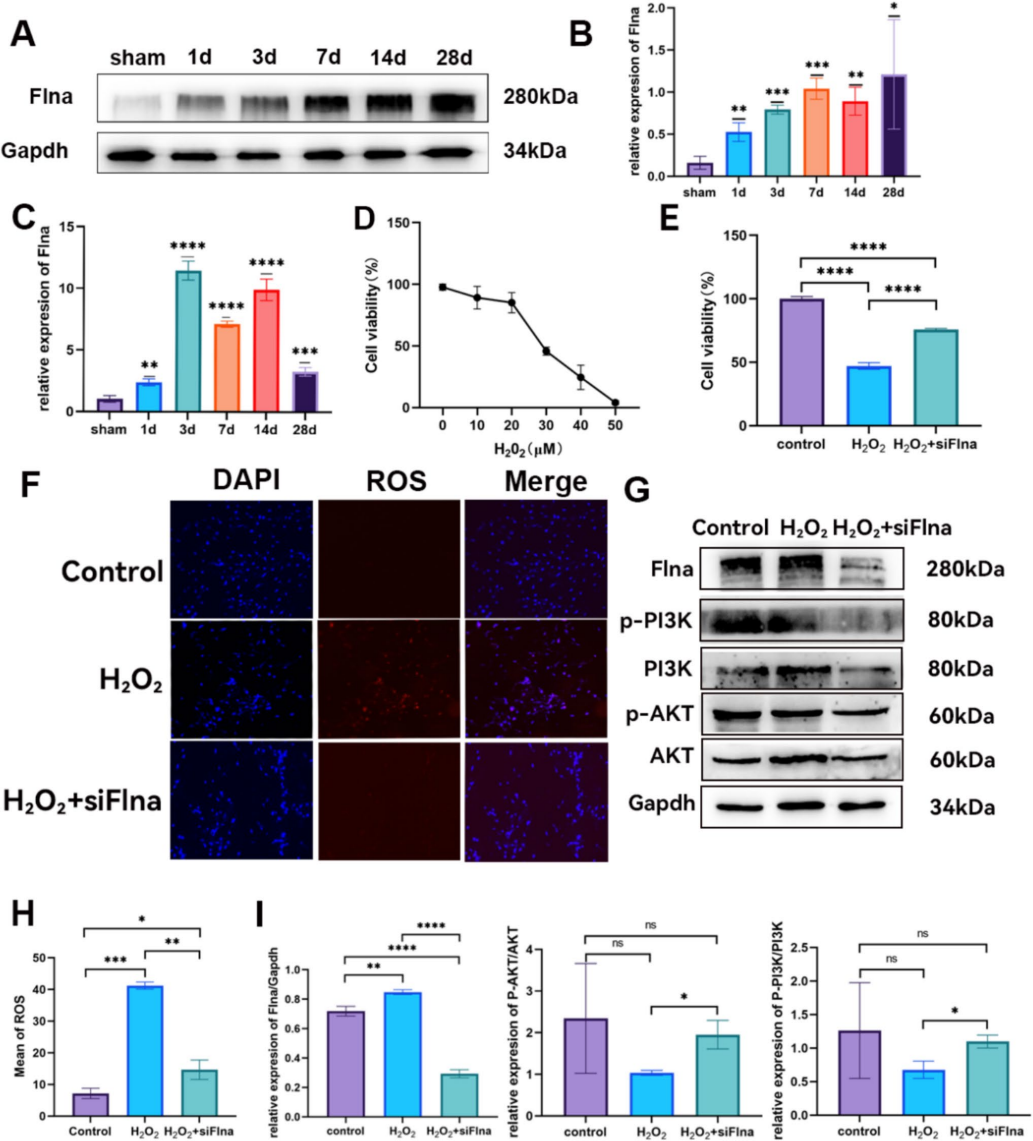
Expression and function of FLNA after SCI. (**A**) FLNA protein expression in the sham group and SCI model animal group on days 1, 3, 7, 14, and 28. (**B**) Histogram of the data from panel A. (**C**) qRT‒PCR detection of FLNA mRNA expression in the sham group and SCI model animal group on days 1, 3, 7, 14, and 28. (**D**) Determination of the half-lethal concentration (IC50) of H_2_O_2_. (**E**) Histogram of the results of the statistical analysis of cell viability in each group via a CCK-8 assay. (**F**) Determination of ROS levels in each group using the DHE method. (**H**) Histogram of the average fluorescence intensity from panel F. (**G**) WB detection of the expression of PI3K and AKT in the PI3K/AKT signalling pathway. (**I**) Histogram of the results of the statistical analysis of key proteins in the PI3K/AKT signalling pathway; * *P* < 0.05, ** *P* < 0.01, *** *P* < 0.0001, ***** *P* < 0.001, ns indicates *P* > 0.05

To further elucidate the expression characteristics and biological functions of FLNA in SCI patients, we conducted subsequent experiments in rats and PC12 cells. First, WB and qRT‒PCR experiments confirmed that the protein and mRNA expression of FLNA was significantly greater in the SCI group rats than in those of the sham group (Fig. 8A–C). Next, to construct an in vitro cell injury model, we treated PC12 cells with a half-lethal concentration of H₂O₂. The results demonstrated that the 24-h half-lethal concentration of H_2_O_2_-treated PC12 cells was 30 µmol/L (Fig. 8D). Subsequently, FLNA expression in PC12 cells was silenced using siRNA, and the interference efficiency was assessed by WB (Supplementary Fig. 1). The results of a CCK-8 assay revealed that, compared with that in the H₂O₂ treatment group, the apoptosis rate of the H₂O₂ + siFLNA group was significantly lower (Fig. 8E). Further examination of the intracellular ROS levels revealed that H₂O₂ injury significantly increased ROS levels, indicating mitochondrial dysfunction. However, after FLNA expression knockdown, the intracellular ROS levels significantly decreased (Fig. 8F, H). Finally, to explore the molecular mechanisms by which FLNA expression regulates mitochondrial injury, we examined the expression levels of key proteins in the PI3K/AKT signalling pathway. H₂O₂ treatment significantly inhibited the expression of PI3K/AKT, whereas FLNA expression knockdown activated the PI3K/AKT signalling pathway, suggesting that FLNA expression may alleviate mitochondrial injury in neurons after SCI by regulating the PI3K/AKT pathway (Fig. 8G, I).

## Discussion

SCI is a severe challenge in the field of central nervous system diseases, with pathological features including neuronal apoptosis, axonal demyelination, and the formation of glial scars (Gao et al. 2024).The pathophysiological mechanisms of SCI involve dysfunction of multiple systems, including the nervous, immune, respiratory, and circulatory systems (Yao et al. 2022; Hashimoto et al. 2024). On the basis of the progression of injury, SCI can be classified into primary and secondary injuries. Specifically, during the acute or subacute phase of secondary injury, the excessive production of ROS triggers oxidative stress, which significantly reduces neuronal activity, thereby hindering the process of neuronal regeneration (Yin et al. 2024b). Oxidative stress occurs when ROS production exceeds the capacity of the body’s antioxidant defence system. Owing to excessive ROS generation, insufficient antioxidant defence capacity, and limited tissue regeneration ability, the body’s defence system is compromised, ultimately leading to neuronal death (Hou et al. 2025). Mitochondria, the core organelles of oxidative metabolism, are critical for maintaining redox homeostasis (Yao et al. 2023). They also play a key, multifaceted role in various pathological processes of SCI. (1) Excitotoxicity and reactive substances are the main triggers of cell death after SCI, leading to mitochondrial dysfunction in neurons and glial cells; (2) cellular metabolic activities influence the inflammatory process by regulating the phenotypes and responses of immune cells; and (3) changes in the number and location of mitochondria are closely related to axonal regeneration and the development of neural stem cells (Slater et al. 2022). The impact of ROS production driven by mitochondrial dysfunction on neuronal damage has been widely studied (Torregrossa et al. 2020). These pathological processes not only lead to immediate loss of motor and sensory function but also severely hinder neurogenesis and functional recovery. Therefore, mitochondrial function in neurons is crucial for functional recovery after injury, but the complex molecular mechanisms that control neuronal survival after SCI remain to be fully elucidated. In this study, we aimed to identify DEGs associated with SCI, identify potential biomarkers and therapeutic targets, and provide a novel theoretical basis for improving neuronal survival and promoting functional recovery.

In this study, we systematically screened key regulatory proteins associated with SCI by combining animal experiments and proteomics analysis methods. By comparing the differentially expressed proteins at different time points after SCI with those in the sham surgery group, a total of 351 DEPs were identified, including 92 upregulated proteins and 259 downregulated proteins. Subsequent analysis via the WGCNA method revealed 14 relevant modules. By combining these modules with the previously identified 351 DEPs, we selected a final set of 42 differentially expressed proteins with common expression changes. To identify potential biomarkers for SCI accurately in rats, two machine learning algorithms were applied for systematic analysis of the 42 differentially expressed proteins. The results revealed that the common intersecting protein identified by both algorithms was FLNA. Notably, the area under the ROC curve (AUC) for the FLNA protein reached 100.0%, indicating its extremely high diagnostic accuracy. To validate the reliability of these findings, further verification was conducted with the independent datasets GSE183591 and GSE45006. The results demonstrated that the AUC of the ROC curve for the FLNA protein was also 100.0% in each independent dataset, providing strong evidence that FLNA has excellent model-differentiation ability across different datasets.

FLNA, a key member of the filamin protein family, is an actin-binding protein encoded by a protein on the X chromosome that plays an essential role in various biological processes (Gorlin et al. 1990). Recent RNA sequencing results have demonstrated that FLNA plays a key regulatory role in the pathological processes associated with SCI, suggesting its potential impact on neural repair and regeneration (Wang et al. 2020). Research has indicated that FLNA interacts with more than 90 different proteins that are widely involved in critical biological processes such as cell signalling, migration, adhesion, receptor activation, and transcriptional regulation (Savoy And Ghosh 2013). By analysing the spatial expression pattern of FLNA through single-cell sequencing data, we found that FLNA is significantly differentially expressed across different cell types, with especially high expression in endothelial cells and neurons. Studies have shown that FLNA regulates the barrier function of endothelial cells through its interaction with RAS (Nallapalli et al. 2012). Furthermore, FLNA is widely expressed in developing cortices and mature pyramidal neurons (Falace et al. 2024). We focused on the role of FLNA in epilepsy, cell migration, cardiovascular development, and increased dendritic complexity. These findings suggest that normal FLNA expression levels are crucial for the proper development of dendrites and that its loss of expression leads to significant changes in dendritic morphology and numbers, indicating that FLNA is a key regulatory protein involved in establishing functional neuronal connections (Zhang et al. 2014). FLNA not only serves as an important component of the cytoskeleton but also plays a key bridge role between receptor signalling pathways and the cytoskeleton, regulating multiple cell functions (Zhang 2025b).In addition, GO and KEGG enrichment analyses revealed that FLNA is involved primarily in protein binding and interactions, with related signalling pathways mainly involving oxidative phosphorylation, neurodegeneration, and cell apoptosis. Notably, our experiments revealed that the protein and RNA expression levels of FLNA are significantly increased after SCI. Although both the protein and mRNA expression levels were elevated, differences were detected. While posttranscriptional regulation (e.g., miRNA-mediated translation suppression) or protein stability changes may explain this phenomenon, the current evidence remains inconclusive. We emphasise this as a limitation and propose further mechanistic studies. Further in vitro experiments revealed that downregulation of FLNA expression alleviated H_2_O_2_-induced damage in PC12 cells, increased cell viability, and reduced intracellular ROS levels. On the basis of the above findings, we speculate that FLNA may significantly promote neuronal survival after SCI.

The KEGG analysis results suggested that FLNA is involved in a complex regulatory relationship with oxidative phosphorylation and cell apoptosis. Among these pathways, the PI3K/AKT signalling pathway is considered a classic pathway that plays a key role in SCI (Xiao et al. 2022; He et al. 2022). Phosphoinositide 3-kinase (PI3K) is a lipid kinase that plays an important role in mediating various physiological and pathological activities within cells. It regulates biological processes such as cell proliferation, differentiation, programmed cell death, and migration by activating its downstream effector molecule, protein kinase B (AKT) (He et al. 2022). Studies have shown that activating the PI3K/AKT signalling pathway can significantly reduce the expression levels of caspase-9 and caspase-3, thus effectively inhibiting cell apoptosis following SCI in rats (Chen et al. 2018). Jung et al. reported that treadmill exercise training could upregulate the expression of neurotrophic factors such as NGF, NT-3, and IGF-1 in rats, thereby activating the PI3K/AKT signalling pathway, inhibiting spinal cord cell apoptosis, and improving motor function after injury (Jung et al. 2014). Additionally, Hu et al. reported that lignans can alleviate neuroinflammation and reduce microglial cell apoptosis after SCI through the PI3K/AKT signalling pathway (Hu et al. 2023). In our study, FLNA expression silencing significantly reduced p-PI3K/PI3K and p-AKT/AKT levels. Therefore, we speculate that silencing FLNA expression may increase the activity of PC12 cells and reduce intracellular ROS levels by regulating the PI3K/AKT signalling pathway. However, the specific molecular mechanism by which FLNA interacts with the PI3K/AKT pathway still requires further investigation.

In summary, in this study, we identified key proteins as diagnostic and therapeutic targets through DEGs and WGCNA, revealing the molecular mechanisms underlying neuronal survival after SCI. Using advanced machine learning methods, including the random forest algorithm and support vector machine recursive feature elimination, we ultimately identified the key protein FLNA. In our dataset, the AUC of FLNA reached 100.0%. To further validate the diagnostic efficacy of this protein, we conducted experiments using the GSE183591 and GSE45006 datasets. The results revealed that the AUC of FLNA was 100.0% in both datasets, demonstrating its strong model discrimination ability across different datasets. To observe the spatial expression pattern of FLNA, we subsequently analysed single-cell sequencing data, which revealed that FLNA is expressed primarily in endothelial cells and neurons. Moreover, the increase in FLNA expression after injury was statistically significant across different datasets. Enrichment analysis indicated that FLNA plays a role in cell survival in H2O2-induced PC12 cell damage. It is associated with increased neuronal apoptosis and oxidative stress. These findings suggest that FLNA expression may exacerbate the SCI process. In PC12 cell experiments, FLNA expression knockdown not only alleviated H2O2-induced cell apoptosis but also significantly reduced ROS production, indicating a protective mechanism. Additionally, the inhibition of the PI3K/AKT signalling pathway following FLNA expression knockdown further suggested its involvement in regulating PC12 cell survival. These findings and analyses contribute to a deeper understanding of the molecular basis of neuronal function after SCI and suggest that targeting FLNA may be a promising strategy for enhancing neuroprotection and promoting recovery following SCI. In addition, FLNA has demonstrated potential for clinical translation in terms of diagnosis and treatment, as it achieved an AUC of 100.0% in diagnosing SCI across multiple datasets, indicating high diagnostic accuracy, and is involved in key pathophysiological processes of SCI, influencing cell survival and apoptosis.

## Supplementary Information

Below is the link to the electronic supplementary material.

Supplementary file1 (XLSX 186 KB)

Supplementary file2 (DOCX 560 KB)

## Data Availability

The analysed datasets generated during the study are available from the corresponding author upon reasonable request.
