# Supplementary figures and images for: Machine Learning Identifies FLNA as a Key Molecular Target Regulating Neuronal Apoptosis after Spinal Cord Injury
Source: J Mol Neurosci. 2025 Nov 15;75(4):151. doi: 10.1007/s12031-025-02439-z (PMC12619718; doi:10.1007/s12031-025-02439-z)

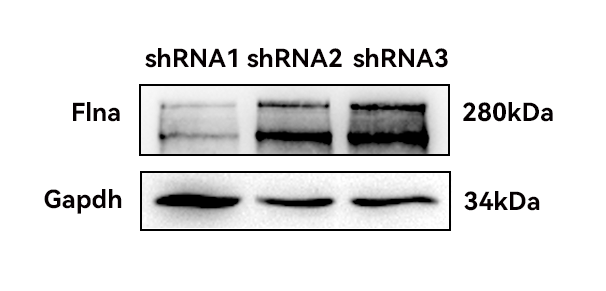


Supplementary Fig. 1 WB analysis revealed low FLNA expression knockdown efficiency in PC12 cells.

Supplement: Supplementary file 2 — Supplementary file2 (DOCX 560 KB) [file 12031_2025_2439_MOESM2_ESM.docx]
